# Microneedle/nanoencapsulation-mediated transdermal delivery: Mechanistic insights

**DOI:** 10.1016/j.ejpb.2013.01.026

**Published:** 2014-02

**Authors:** Yasmine A. Gomaa, Martin J. Garland, Fiona J. McInnes, Ryan F. Donnelly, Labiba K. El-Khordagui, Clive G. Wilson

**Affiliations:** a Strathclyde Institute of Pharmacy and Biomedical Sciences (SIPBS), University of Strathclyde, Scotland, UK; b School of Pharmacy, Queen’s University of Belfast, Northern Ireland, UK; c Department of Pharmaceutics, Faculty of Pharmacy, Alexandria University, Alexandria, Egypt

**Keywords:** MN, microneedle, NP, nanoparticle, Rh B, rhodamine B, FITC, fluorescein isothiocyanate, Microneedles, PLGA nanoparticles, Transdermal delivery, Confocal laser scanning microscopy, Skin permeation, Rhodamine B, Fluorescein isothicoyanate

## Abstract

A systematic study was undertaken to gain more insight into the mechanism of transdermal delivery of nanoencapsulated model dyes across microneedle (MN)-treated skin, a complex process not yet explored. Rhodamine B (Rh B) and fluorescein isothiocyanate (FITC) as model hydrophilic and hydrophobic small/medium-size molecules, respectively, were encapsulated in poly lactic-co-glycolic acid (PLGA) nanoparticles (NPs) and delivered through full thickness porcine skin pretreated with MN array. Permeation through MN-treated skin was affected by physicochemical characteristics of NPs and the encapsulated dyes. Dye flux was enhanced by smaller particle size, hydrophilicity, and negative zeta potential of NPs. Regarding encapsulated dyes, solubility at physiological pH and potential interaction with skin proteins proved to outweigh molecular weight as determinants of skin permeation. Data were verified using confocal laser scanning microscopy imaging. Findings coupled with the literature data are supportive of a mechanism involving influx of NPs, particularly of smaller size, deep into MN-created channels, generating depot dye-rich reservoirs. Molecular diffusion of the released dye across viable skin layers proceeds at a rate determined by its molecular characteristics. Data obtained provide mechanistic information of importance to the development of formulation strategies for more effective intradermal and transdermal MN-mediated delivery of nanoencapsulated therapeutic agents.

## Graphical abstract

Using PLGA nanoparticles (NPs) with similar size and zeta potential, skin permeation of nanocapsulated dyes across microneedle-treated skin is determined by the molecular characteristics. Findings are important for the microneedle-mediated transdermal and dermal delivery of nanocapsulated therapeutic agents.

**Figure:**
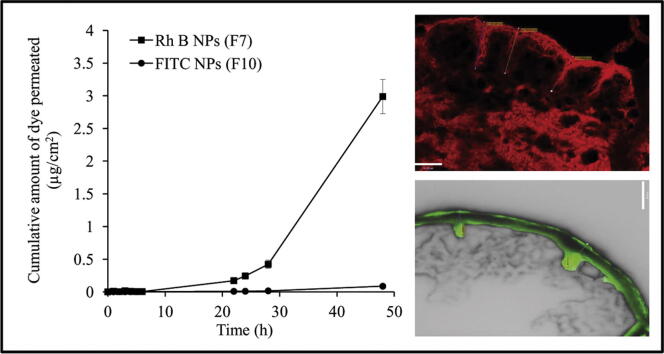

## 1 Introduction

Transdermal delivery of drugs with unfavorable skin absorption using microneedle (MN) array technology has the potential of bringing to clinical practice more effective and safer products [1], [2], [3]. By penetrating the skin in a minimally-invasive manner, native or drug-loaded MNs create microchannels in the *stratum corneum* (*SC*) and epidermis as in-skin pathways for drug diffusion. This permits an increase in several orders of magnitude in the passage or dermal targeting of drugs ranging from small hydrophilic molecules such as alendronate [4] to macromolecules, including low molecular weight heparins [5] insulin [6] and vaccines [7], [8].

While MN-mediated transdermal drug delivery has been extensively investigated, the use of MN technology for transdermal delivery of drug-loaded nanocarriers is novel [9], [10], [11]. An optimized MN/drug-loaded nanocarrier transdermal delivery approach may allow modulation of the absorption of the drug of interest [10]. For example, polymeric nanoparticles (NPs) offer a wide range of benefits including in-skin drug targeting, control of skin permeation, protection of the encapsulated drug from degradation in the biological milieu in addition to reduced dose, and side effects [12]. Drug release from NPs can be modulated by selectively modifying factors associated with shape, size, chemical composition, internal morphology, surface charge, and use of combined enhancing strategies [13], [14], [15]. Without the use of physical methods of skin permeation, the literature reports suggest that in most instances, polymeric NPs penetrate the *SC* poorly [16], [17] following passive routes of permeation through the hair follicles where the drug is released and transported to deeper skin layers [18], [19]. Intuitively, delivering NPs beyond the *SC* with the simultaneous creation of additional larger and denser in-skin pathways would promote translocation of NPs as drug-rich reservoirs deeper into the skin. A combined MN/nanoencapsulation approach has been reported for the intradermal delivery of a nanoencapsulated lipophilic model of a photosensitizer using dissolving MNs [11] and the *in vitro* transdermal delivery of a rhodamine B (Rh B) as a model medium-size dye using the poke and patch technique [10]. More effective exploitation of the approach, however, should be based on a better understanding of the variables controlling translocation of NPs through the aqueous MN-created channels, particularly those involved in in-skin drug release and the concentration gradient-driven diffusion of the released encapsulated species across hydrophilic, viable skin layers [20].

Confocal laser scanning microscopy (CLSM) indicated that penetration and distribution of fluorescent polymeric NPs into MN-treated skin are confined to the hair follicles and MN-created channels in a size and concentration-dependent manner, with significantly denser localization in the epidermis compared to the dermis [21], [22]. However, transdermal delivery of polymer NPs across MN-treated skin has been a matter of controversy. While polystyrene NPs applied to a MN-treated human epidermal membrane reached receptor solutions in permeation experiments [23], [24], poly lactic-co-glycolic (PLGA) NPs could not permeate full thickness human abdominal skin [22], murine [21], or porcine ear skin [10].

In a recent study [10], we related MN characteristics and application variables to the *in vitro* skin permeation of a nanoencapsulated medium-size dye, Rh B, across MN-treated full thickness porcine skin. In the present study, more insight into the mechanism of MN-driven skin permeation of nanoencapsulated dyes as model drugs was sought. The contribution of the carrier and encapsulated dye characteristics to MN-mediated skin permeation was investigated using PLGA NPs with different physicochemical attributes and Rh B and fluorescein isothiocyanate (FITC) as model hydrophilic and hydrophobic molecules, respectively [25]. Both dyes are easily determined spectrofluorometrically [26] and have been widely used in fluorescence-based imaging applications [19], [27], [28]. Further, the two dyes were used in an earlier report [25] to examine possible correlation of molecular characteristics with passive diffusion and MN-mediated permeation through full thickness porcine skin.

## 2 Material and methods

### 2.1 Chemicals

Poly lactic-co-glycolic acid (PLGA), Resomer RG 503 H (50:50) (MW 24,000–38,000 Da), and Resomer RG 753 S (75:25) (MW 36,610 Da) both of inherent viscosity of 0.32–0.44 dl/g in 0.1% in chloroform at 25 °C and Polylactic acid (PLA) Resomer R 203 H (MW 18,000–28,000 Da) of inherent viscosity 0.25–0.35 dl/g were purchased from Boehringer Ingelheim (Ingelheim, Germany). Rhodamine B (Rh B, MW 479.02 Da), fluorescein isothiocyanate (FITC, MW 389.38 Da), Didodecyldimethyl ammonium bromide (DMAB), Polyvinyl alcohol (PVA, MW 30–70 kDa), and phosphate buffer saline (PBS) tablets (pH 7.4) were obtained from Sigma–Aldrich (St. Louis, MO, USA). Ethyl acetate, AR grade (Fisher Scientific UK Ltd., Loughborough, UK), Nanovan®, methylamine vanadate stain (Nanoprobes®, Nanophank, NY, USA) “Silver dag” – a colloidal silver preparation – (Polysciences Inc., Eppelheim, Germany) and Silastic® 9280/60E silicone elastomer (Dow Corning, Midland, MI, USA) were also used. Gantrez® AN-139, a copolymer of methylvinylether and maleic anhydride (PMVE/MA), was a gift provided by Ashland (Waterfield Tadworth Surrey, KT20 5HQ, UK). Shandon M-1 embedding OCT (optimal cutting temperature) matrix was purchased from Thermo Electron Corporation (Beenham, Reading, UK).

### 2.2 Preparation and characterization of Rh B and FITC-loaded PLGA nanoparticles

#### 2.2.1 Preparation of nanoparticles (NPs)

NPs were prepared using a modified emulsion–diffusion–evaporation method used in an earlier study where reproducibility of dye content, size, and surface charge of Rh B-loaded PLGA NPs has been demonstrated using triplicate experiments [10]. In brief, 50 mg of polymer was dissolved in 2.5 mL ethyl acetate for 2 h at ambient temperature using a magnetic stirrer (Cimarec i Poly 15 Multipoint stirrer, Thermo Electron Corporation, Beenham, Reading, UK). For the preparation of Rh B-loaded NPs, a 200 μL aliquot of an aqueous Rh B solution of specified concentration was emulsified in the organic phase for 5 min using a high speed homogenizer (Polytron PT4000, Littau, Switzerland) to produce a w/o emulsion. An aqueous DMAB solution (5 mL) of specified concentration was added to the resulting emulsion under stirring to produce a w/o/w emulsion. This was followed by homogenization for 5 min. The resulting emulsion was diluted with 25 mL of water with constant stirring. For FITC-loaded NPs, specified weights of the dye were dissolved in the polymer solution prior to the addition of either PVA or DMAB solution of specified concentration, followed by a single homogenization step to yield an o/w emulsion. This was diluted with water (25 mL) and stirred to allow solvent evaporation.

Selected formulation variables and the emulsion homogenization speed were modulated to generate dye-loaded PLGA NPs with different physicochemical characteristics (NPs size, hydrophilicity, surface charge, dye type, and dye initial loading). NPs size was modified by controlling the emulsion homogenization speed (5000, 10,000 and 15,000 rpm), while NPs hydrophilicity was modulated using PLGA copolymer with different lactic to glycolic acid ratios (50:50, 75:25, 100:0). The type of NPs surface charge was determined by the emulsion stabilizer used. DMAB resulted in positively charged NPs, while PVA produced negatively charged NPs. The dye loading of NPs dispersions with Rh B and FITC was increased by adjusting the initial loading (5%, 10%, and 20% w/w) during emulsification. Unless otherwise mentioned, all experiments were conducted by varying one parameter while keeping other parameters set at selected conditions. Table 1 shows the test dye-loaded NP formulations obtained by modulating formulation variables and homogenization speed.

**Table 1:** Formulation variables and pharmaceutical attributes of PLGA NPs prepared using an emulsion–diffusion–evaporation method.

Dye	Formula code	NPs formulation variables				Homogenization speed, rpm	NPs properties, mean ± SD (*n* ⩾ 5)		
		PLGA composition	Dye (% w/w)	DMAB (% w/v)	PVA (% w/v)		Size (nm)	PDI	Zeta potential (mV)
Rh B	F1	50:50	20	3	–	5000	422.3 ± 5.52	0.37 ± 0.04	65.7 ± 1.53
	F2	50:50	20	3	–	10,000	251.5 ± 7.80	0.67 ± 0.06	67.6 ± 1.19
	F3	50:50	20	3	–	15,000	155.2 ± 4.73	0.48 ± 0.02	66.4 ± 1.51
	F4	100:0	20	1	–	15,000	91.90 ± 2.47	0.17 ± 0.01	54.7 ± 2.37
	F5	75:25	20	1	–	15,000	101.1 ± 2.90	0.15 ± 0.02	50.4 ± 1.52
	F6	50:50	20	1	–	15,000	105.5 ± 2.92	0.15 ± 0.02	55.2 ± 2.86
	F7	50:50	10	1	–	15,000	117.4 ± 0.94	0.16 ± 0.01	57.0 ± 1.21
	F8	50:50	5	1	–	15,000	121.7 ± 3.13	0.15 ± 0.02	54.1 ± 1.44

FITC	F9	50:50	5	1	–	15,000	130.9 ± 4.85	0.16 ± 0.01	66.1 ± 2.28
	F10	50:50	10	1	–	15,000	122.0 ± 3.15	0.15 ± 0.02	57.1 ± 1.51
	F11	50:50	20	1	–	15,000	362.7 ± 78.48	0.67 ± 0.04	36.1 ± 2.09
	F12	50:50	10	–	1	15,000	367.0 ± 22.46	0.41 ± 0.05	−4.5 ± 0.45

#### 2.2.2 Transmission electron microscopy (TEM) of NPs

The morphology of NPs was examined by transmission electron microscopy (TEM) (LEO 912 AB Omega, Zeiss, Oberkochen, Germany). A 50 μL volume of diluted NP dispersion (1:10) was placed onto the surface of a formvar/carbon coated 300 mesh grid and allowed to settle for 30 s. Excess sample was drained from the grid by touching the edge with a filter paper. A 5 μL volume of Nanovan® was then added to the sample and removed immediately afterward. The grids were left to dry and examined using TEM.

#### 2.2.3 Determination of the size and zeta potential of NPs

The size and size distribution (polydispersity index, PDI) of the NPs was determined by photon correlation spectroscopy using a Zetasizer (Nano ZS dynamic light scattering instrument, Malvern Instruments Ltd., Malvern, UK). Each sample was run five times. The same instrument was used to determine the zeta potential values of the NPs dispersed in distilled water. Each determination represented a mean value derived from 30 replicate measurements.

#### 2.2.4 Determination of Rh B and FITC content of NPs dispersions

The fluorescence of NP dispersion samples diluted with PBS (pH 7.4) was determined by fluorescence spectrophotometry as reported [26]. The fluorescence intensity of a 300-fold diluted translucent sample of the prepared NP dispersion was measured using a Varian Cary Eclipse fluorescence spectrophotometer (Varian Australia Pty Ltd., Mulgrave, Victoria, Australia). The excitation/emission wavelengths were set to 540/625 and 495/525 nm for Rh B and FITC, respectively.

#### 2.2.5 *In vitro* release of Rh B NPs

A 500 μL-sample of Rh B NPs dispersions of different PLGA composition (F3, F4 and F5) was placed in 1 mL ready-to-use dialysis devices (Float-A-Lyzer® G2, 20 kDa MWCO, Spectra/Por®, USA). Prior to use, the screw caps were removed, and the devices were submerged open and allowed to soak in deionized water for 30 min to remove the impregnating glycerol added by the manufacturer for protection. The devices were allowed to float vertically using the floatation rings at 37 °C in a 10 mL-beaker containing 8 mL of PBS pH 7.4, selected to correlate release data with skin permeation data. The release medium was stirred using small magnetic bars at 500 rpm and a multipoint magnetic stirrer (Cimarec i Poly 15 Multipoint stirrer, Thermo Electron Corporation, Beenham, Reading, UK). Samples (100 μL each) were removed from the beakers at specified time intervals for up to 6 h. An equal volume of fresh PBS (pH 7.4) was added to maintain a constant volume. The withdrawn samples were analyzed by fluorescence spectroscopy as described earlier.

### 2.3 Fabrication and imaging of microneedle (MN) arrays

MN arrays were fabricated using 30% w/v aqueous polymeric solution of PMVE/MA copolymer and laser-engineered silicone micro-molding, as described previously [29], [30]. For scanning electron microscopy (SEM) imaging, arrays were mounted on aluminum stubs using double-sided adhesive tape and “silver dag.” A SC515 SEM sputter coater (Polaron, East Grinstead, UK) was used to coat the arrays with a 20 nm-thick layer of gold/palladium. The arrays were observed under a JSM 6400 digital SEM (JEOL Ltd., Tokyo, Japan), and photomicrographs of MN structures were obtained.

### 2.4 *In vitro* skin permeation studies

Full thickness porcine skin was obtained from ears of pigs (Landrace species), harvested immediately following slaughter at a local abattoir (Glasgow, UK). The ears were sectioned using a scalpel to yield whole skin samples. The average thickness of skin samples, as measured using a digital micrometer, was 1164 ± 103 μm (*n* = 46). MN arrays of 600 μm length, 121 MNs/array in density (11 × 11) were manually pressed onto the center of each skin sample five times, and MN arrays were rotated ∼ 90° before each re-insertion. The last insertion of the MN lasted 2 s before retraction of the array. The MN-treated skin samples were inserted as barrier membranes in the Franz diffusion cells (PermeGear, Bethlehem, PA, USA). These were attached to thermostatically-modulated water pump (Haake DC10, Karlsruhe, Germany). The receiver cells contained 5.3 mL PBS (pH 7.4), which was stirred at 600 rpm and maintained at 37 ± 0.5 °C. Skin samples were initially left in the Franz cells for 1 h to allow for hydration. The permeation experiment was started by adding a 500 μL aliquot of test NP formulation onto each skin sample. The dye content of test NP formulations was adjusted to 77.5 μg/mL by diluting the final NP dispersion with distilled water [26] leading to a constant dye content but variable NP concentration. The effect of NPs size, PLGA copolymer ratio, surface charge, dye type, and % of initial dye loading on *in vitro* permeation through MN-treated porcine skin was investigated. FITC NPs with positive and negative zeta potential were used to test the effect of surface charge on skin permeation of the nanoencapsulated dye.

In all cases, a 100 μL-sample was removed from the sampling arm at specific intervals over 48 h, while an equal volume of fresh PBS was added to maintain a constant volume. The withdrawn samples were analyzed by fluorescence spectroscopy as mentioned earlier taking into account the progressive dilution of the receiver phase occurring over the course of the experiment. The cumulative amount of dye permeating through the skin was plotted as a function of time. The steady state flux was calculated as the slope of the linear portion of their time permeation profile divided by the diffusional area (0.64 cm^2^) of the skin sample. Data presented are the mean of at least three experiments.

### 2.5 Confocal laser scanning microscopy (CLSM)

At the end of the permeation experiment, skin samples exposed to Rh B NPs (F7) and FITC NPs (F10) were collected and the *SC* cleaned thoroughly under running cold water then blotted dry with soft tissue. For viewing vertical skin sections, skin was embedded in OCT medium and cryo-sectioned to 10 μm-thick vertical sections using a Shandon Cryotome® (SME Cryostat, Fisher Thermo Scientific, Asheville, NC, USA). Same sectioning technique was used in order to obtain relative results. Transmission images of the skin were recorded using a Leica TCSP5 confocal microscope connected to a DM6000B upright microscope (Leica Microsystems GmbH, Wetzlar, Germany) with an HCX-APO-L-U-V-1 20× 0.5 water dipping objective in case of *Z*-stacks of full thickness or a 20× Leica HC.PL. Fluotar (dry) objective (0.5 NA) in case of vertical skin sections. Excitation was provided by a 514 nm (Rh B NPs, F7) and 488 nm (FITC NPs, F10) Argon laser (20 mW) line with filtered-base emission channels of 565–625 nm (F7) and 491–567 nm (F10). *X*–*Z* sectioning was performed to detect dye depth of penetration. For viewing *Z*-stacks of full skin thickness, the *Z*-axis images were gathered at 10 μm planes to a total depth of 200 μm using the 543 nm Argon laser line set to 40% of output. The frame size was set to 1024 × 1024 pixels, and the image was composed of 3 frames. Gain and offset were maximized to enhance contrast. Subsequent image visualization was performed using High Performance 3D–4D imaging software (Volocity 5.5, Improvision). The depth of the microchannels was estimated indirectly based on the depth of dye permeation.

### 2.6 Statistical analysis

Where appropriate, a Mann–Whitney *U* or a Kruskal–Wallis test followed by a *post hoc* Dunn’s test was used to analyze permeation data using SPSS software (SPSS Inc., Chicago, IL, USA). In all cases, *P* ⩽ 0.05 denoted significance.

## 3 Results

**Fig. 1:**
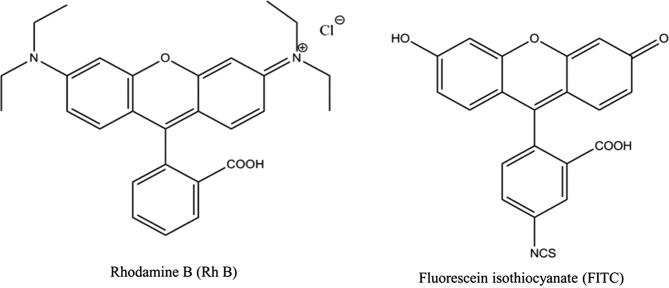
Chemical structures of the two dyes selected for the study.

**Fig. 2:**
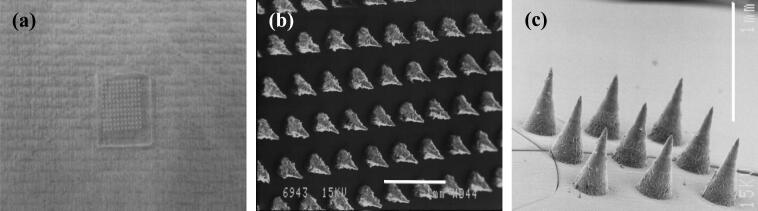
Digital photographs of polymer MN arrays with 600 μm long MNs and a density of 121 MNs/array (a). SEM images of MN arrays showing top (b) and side (c) views. Bar scales represent 1 mm.

The study involved assessment of the effect of characteristics of PLGA NPs (size, hydrophilicity, and charge) and dyes encapsulated therein (molecular weight, solubility, and % initial loading) on skin permeation using the dual MN/nanoencapsulation approach. The structures of the two dyes used in the study (Rh B and FITC) are shown in Fig. 1. At physiological pH, Rh B is zwitterionic with a net neutral charge, while FITC is anionic [25]. The design of polymer MN arrays and application mode used in this study was based on data reported earlier for the effect of MN characteristics on *in vitro* skin permeation of nanoencapsulated Rh B [10]. As shown in Fig. 2, MNs were conical in shape, with an average basal width of 300 μm, an average length of 600 μm and arranged at an inter-needle spacing of 300 μm with a density of 121 MNs per array.

### 3.1 PLGA NPs with different physicochemical characteristics

PLGA NPs with controlled physicochemical properties were prepared using 2% w/v polymer and an emulsion–solvent evaporation method [10] with modulation of formulation variables and homogenization speeds (Table 1). The variable levels were optimized in order to modulate a target property without appreciably affecting other dependent properties. A total of eight Rh B and four FITC test NP formulations were used (Table 1). NPs prepared with DMAB (F1–F11) had a positive zeta potential due to adsorption of the cationic emulsion stabilizer, while those prepared with PVA (F12) had a negative surface charge conferred by the free end carboxylic groups of PLGA. Positive zeta potential values were generally greater than 30 mV.

Table 1 shows that Rh B-loaded PLGA 50:50 NPs (F1–F3) with different size (422.3–155.2 nm) could be obtained using 3% w/v DMAB by increasing emulsion homogenization speed while keeping other formulation variables constant. Further, modulation of NPs hydrophilicity (F4–F6) was achieved by using PLGA with different lactide to glycolide ratio (100:0, 75:25, and 50:50) without discernibly affecting particle size, PDI, and zeta potential of NPs. The table also shows that PLGA 50:50 NPs could be loaded with Rh B at increasing levels (5%, 10%, and 20% w/w, F6–F8) with no detectable effect on particle size, PDI, and zeta potential values. However, increasing FITC loading (F9–F11) particularly at the 20% w/w level was associated with a marked increase in particle size and PDI and reduced zeta potential. The FITC NPs formulation (F12) prepared using 1% w/v PVA as stabilizer showed a zeta potential of −4.5 and a distinct increase in particle size.

**Fig. 3:**
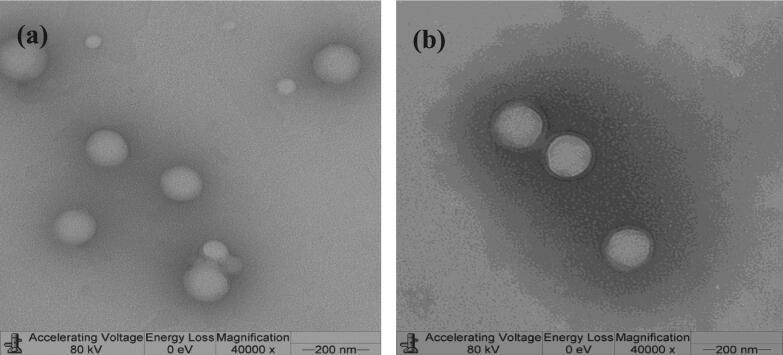
Representative TEM images of Rh B (F8, a) *versus* FITC (F9, b) PLGA 50:50 NPs at 5% w/w initial dye loading.

Fig. 3 shows TEM images of representative Rh B (F8) and FITC (F9) NPs samples prepared using PLGA 50:50 at 5% w/w dye loading. NPs were spherical in shape with more or less uniform size verifying size data presented in Table 1.

### 3.2 *In vitro* skin permeation studies

Data for skin permeation of nanoencapsulated dyes across MN-treated porcine ear skin, expressed as cumulative amount of dye permeating at 48 h (*Q*_48_, μg/cm^2^) and steady state flux (μg/cm^2^/h), are presented in Table 2. Several reports provided evidence for maintenance of the barrier function of porcine skin for up to 48 h [10], [31]. Further, frequent sampling was essential for the initial part of the study due to the lack of the literature data regarding the permeation of a dye loaded into nanoparticles through MN-treated skin.

**Table 2:** Effect of formulation variables on the permeation of nanoencapsulated Rh B (F1–F8) and FITC (F9–F12) across MN-treated full thickness porcine skin. Values are mean ± SD (*n* ⩾ 3).

Dye	Formula code	*Q*_48_ (μg/cm^2^)	Steady state flux (μg/cm^2^/h)
Rh B	F1	0.50 ± 0.20	0.81 ± 0.28
	F2	2.02 ± 0.11	2.83 ± 0.19
	F3	2.49 ± 0.08	3.55 ± 0.09
	F4	2.07 ± 0.19	2.90 ± 0.27
	F5	2.92 ± 1.32	3.98 ± 1.62
	F6	5.40 ± 0.39	6.19 ± 0.77
	F7	2.99 ± 0.26	4.29 ± 0.42
	F8	1.78 ± 0.63	2.53 ± 0.87

FITC	F9	0.13 ± 0.04	0.17 ± 0.05
	F10	0.09 ± 0.01	0.12 ± 0.02
	F11	0.06 ± 0.02	0.09 ± 0.03
	F12	0.24 ± 0.08	0.35 ± 0.11

#### 3.2.1 Effect of NPs-related variables on skin permeation of the nanoencapsulated dye

##### 3.2.1.1 Effect of NPs size

**Fig. 4:**
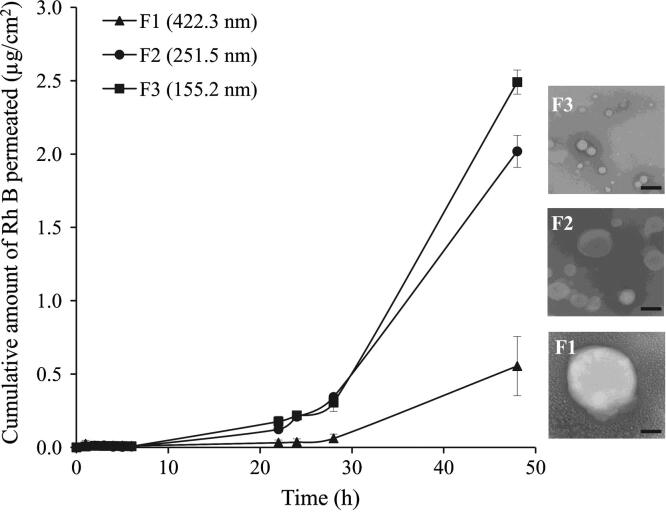
Effect of NPs size on Rh B permeation through MN-treated full thickness porcine skin. Data points shown are mean ± SD (*n* = 3). SEM images of tested NP formulations are shown on the right-hand side of the graph. Bar scale represents 200 nm.

At the 1% w/v DMAB concentration used throughout the study, NPs had a mean diameter of approximately 100 nm (Table 1) which did not noticeably change in response to homogenization speed (screening data not shown). The higher concentrated 3% w/v DMAB solution had a higher viscosity (20.8 ± 0.0026 cP) as measured using a cone and plate viscometer (CSL2 100, TA Instruments, Crawley, UK) compared to that of the 1% w/v solution (3.71 ± 0.0004 cP). It resulted in a measurable increase in particle size that was inversely proportional to the homogenization speed. Thus, NP size was controlled by optimizing emulsion homogenization speed and DMAB concentration (Table 1). The increase in particle size of Rh B-loaded PLGA 50:50 NPs significantly (*P* < 0.05) reduced Rh B skin permeation (Fig. 4) despite the PDI values exceeding 0.2. Mean *Q*_48_ values of 2.49 ± 0.08, 2.02 ± 0.11 and 0.5 ± 0.20 μg/cm^2^ and flux values of 3.55 ± 0.09, 2.83 ± 0.19 and 0.81 ± 0.28 μg/cm^2^/h were obtained for test NPs formulations F1 (155.2 nm), F2 (251.5 nm) and F3 (422.3 nm), respectively.

##### 3.2.1.2 Effect of NPs polymer composition and hydrophilicity

**Fig. 5:**
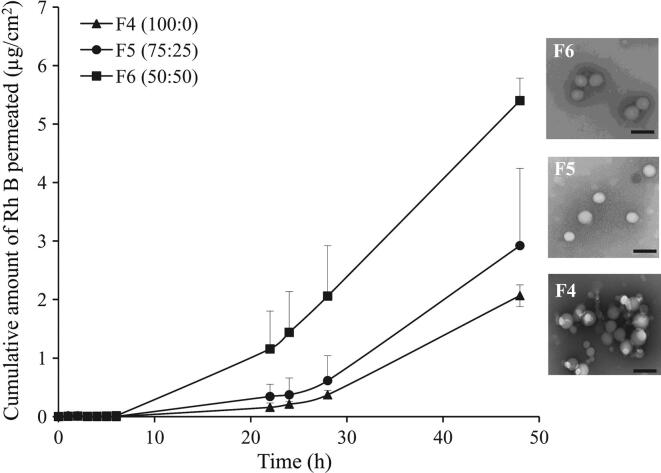
Effect of PLGA nanoparticles hydrophilicity on the permeation of Rh B through MN-treated full thickness porcine skin. Data points shown are mean ± SD (*n* = 3). SEM images of tested NP formulations are shown on the right-hand side of the graph. Bar scale represents 200 nm.

The increase in hydrophilicity of Rh B-loaded PLGA NPs (F4–F6) of more or less similar size (91.9–105.5 nm), achieved by reducing lactide to glycolide ratio, enhanced dye permeation across MN-treated skin (Fig. 5). Data in Table 2 indicated that exposure of skin samples to F4 NPs (PLGA 100:0) resulted in a mean *Q*_48_ of 2.07 ± 0.19 μg/cm^2^ and flux of 2.90 ± 0.27 μg/cm^2^/h. Reducing the lactide to glycolide ratio to 75:25 (F5) increased *Q*_48_ (2.92 ± 1.32 μg/cm^2^) and the flux (3.98 ± 1.62 μg/cm^2^/h) yet not significantly (*P* = 0.379, 0.395, respectively). A further reduction in the lactide content (50:50, F6) caused a significant increase in mean *Q*_48_ (5.40 ± 0.39 μg/cm^2^, *P* = 0.016) with no significant increase in flux (6.19 ± 0.77 μg/cm^2^/h, *P* = 0.072).

**Fig. 6:**
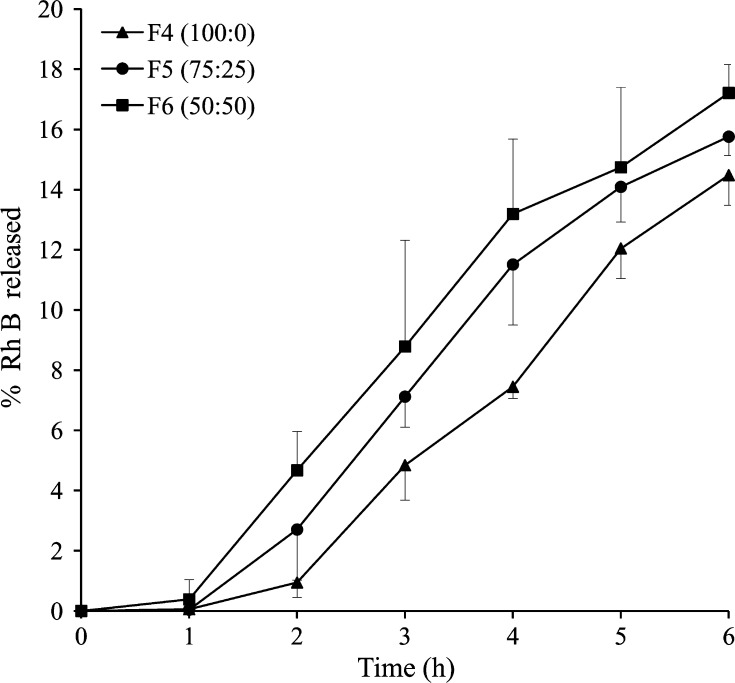
*In vitro* release profiles of Rh B loaded in PLGA NPs of different lactide to glycolide copolymer ratio, (100:0, F4), (75:25, F5) and (50:50, F6) in PBS pH 7.4 at 37 °C. Data points shown are mean ± SD (*n* = 3).

*In vitro* release of Rh B from the three test NPs (F4–F6) at physiological pH indicated enhanced drug release as a function of NPs hydrophilicity (Fig. 6). Release profiles were characterized by lack of burst effect and relatively low release rate indicating efficient dye entrapment. Approximately 14.5%, 15.8%, and 17.2% of the dye was released at 6 h from NPs prepared using PLGA with copolymer ratio of 100:0 (F4), 75:25 (F5), and 50:50 (F6), respectively.

##### 3.2.1.3 Effect of NPs surface charge

**Fig. 7:**
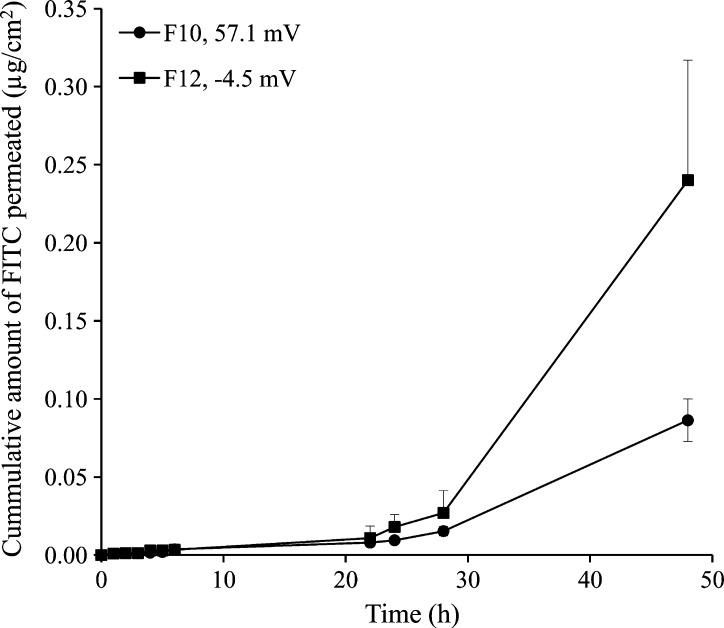
Effect of NPs charge on the permeation of FITC through MN-treated full thickness porcine skin. Data points shown are mean ± SD (*n* = 3).

FITC NPs with positive and negative zeta potential at 10% w/w loading (F10 and F12, respectively) were used. Exposure of skin samples to negatively charged NPs resulted in greater skin permeation of FITC despite the larger NPs size (367.0 *versus* 122.0 nm for F10 and F12, respectively, Fig. 7 and Table 1). The mean *Q*_48_ and flux values for F12 NPs were 0.24 ± 0.08 μg/cm^2^ and 0.35 ± 0.11 μg/cm^2^/h, respectively (Table 2). These corresponded to mean *Q*_48_ and flux values of 0.09 ± 0.01 μg/cm^2^ and 0.12 ± 0.02 μg/cm^2^/h for the positively charged FITC NPs (F10), respectively. Differences between *Q*_48_ and flux values for F10 and F12 were statistically significant (*P* < 0.05).

#### 3.2.2 Effect of encapsulated dye-related variables on skin permeation

**Fig. 8:**
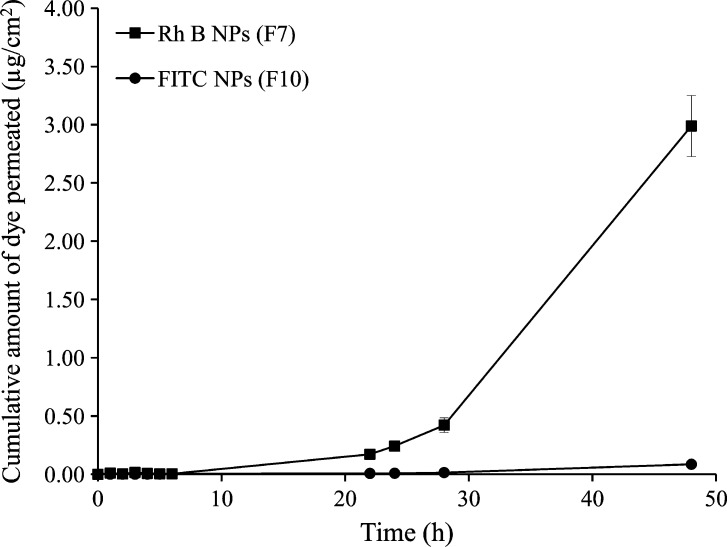
Comparative skin permeation profiles of Rh B (F7) and FITC (F10) NPs at 10% w/w initial dye loading. Data points shown are mean ± SD (*n* = 3).

Fig. 8 shows permeation profiles for Rh B and FITC encapsulated in 50:50 PLGA NPs at 10% w/w loading (F7 and F10, respectively, Table 1). Both formulations had similar particulate properties in terms of size (117.4 and 122.0 nm, respectively) and zeta potential (57 mV). Poorer permeation of FITC was observed with a significantly longer lag period (∼30 h) compared to Rh B NPs (∼6 h), suggesting a different permeation mechanism. A statistically significant 33.2-fold and 35.8-fold difference in *Q*_48_ and flux values, respectively, was observed for Rh B compared to FITC. The *Q*_48_ and flux values for Rh B were 2.99 ± 0.26 μg/cm^2^ and 4.29 ± 0.42 μg/cm^2^/h, respectively. Significantly lower values *(P* < 0.05) for *Q*_48_ (0.09 ± 0.01 μg/cm^2^) and flux (0.12 ± 0.02 μg/cm^2^/h) were obtained for FITC.

**Fig. 9:**
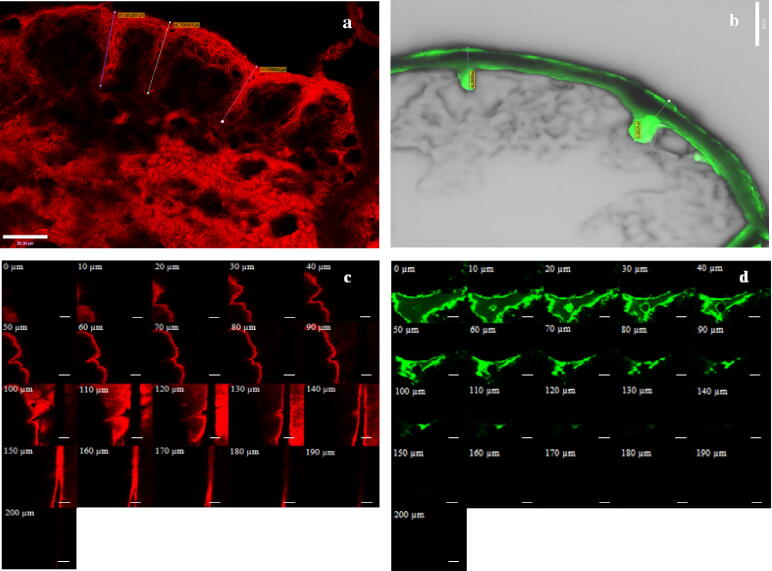
Confocal images of vertical sections (a and b) and *Z*-stacks (c and d) of full thickness porcine skin treated with Rh B (F7) and FITC (F10) PLGA NPs, respectively. Bar scales represent 50 μm (a and b) and 100 μm (c and d). (For interpretation of the references to color in this figure legend, the reader is referred to the web version of this article.)

CLSM images of MN-treated porcine skin exposed to these two NP formulations (F7 and F10) for 48 h were obtained for both vertical sections (surface view of mechanically sectioned skin) and *Z*-stacks to determine the depth of dye permeation (Fig. 9a–d). Rh B and FITC NPs applied to the MN-treated skin surface infiltrated the microchannels as evidenced by the red and green intense fluorescence in Fig. 9a and b, respectively, with deeper penetration of Rh B. Individual NPs could not be visualized as their size was below the resolution limit of the confocal microscope [32], [33]. This is in addition to deterioration of the resolution in real-case scenarios when imaging biological specimens, skin in this case, in which the light suffers several effects such as scattering [34]. While Rh B diffused laterally as indicated by red fluorescence around microchannels and in deeper skin layers (Fig. 9a), FITC fluorescence was mainly restricted to microchannels (Fig. 9b). Penetration depth profiles (*Z*-stacks, Fig. 9c and d) showed deeper Rh B fluorescence, thought to be a result of the released dye reaching a depth of 190 μm compared to 130 μm for FITC. Confocal imaging verified the significantly lower skin permeability of nanoencapsulated FITC compared to Rh B.

**Fig. 10:**
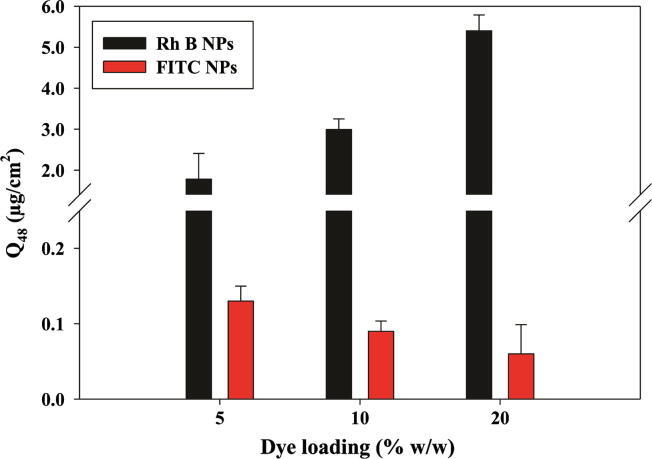
Effect of NPs initial dye loading on cumulative permeation at 48 h (*Q*_48_) through MN-treated full thickness porcine skin. Data points shown are mean ± SD (*n* = 3). (For interpretation of the references to color in this figure legend, the reader is referred to the web version of this article.)

The effect of % initial loading on skin permeation of nanoencapsulated Rh B and FITC is shown in Fig. 10. Transdermal delivery of Rh B increased significantly (*P* < 0.05) with the increase in dye loading. For 5% Rh B loading (F8), *Q*_48_ and flux values were 1.78 ± 0.63 μg/cm^2^ and 2.53 ± 0.87 μg/cm^2^/h, respectively. Increasing loading to 10% w/w (F7) and 20% w/w (F6) caused a significant increase (*P* < 0.05) in both *Q*_48_ (2.99 ± 0.26 and 5.40 ± 0.39 μg/cm^2^, respectively) and flux (4.29 ± 0.42 and 6.19 ± 0.77 μg/cm^2^/h, respectively). Differences between *Q*_48_ and flux values obtained at 10% w/w and 20% w/w initial load were also statistically significant (*P* = 0.001 and 0.030, respectively). On the other hand, increasing initial% FITC loading (5%, 10% and 20% w/w, F9, F10, and F11, respectively, Table 1) led to reduced skin permeation (Fig. 10 and Table 2). NP formulations F9, F10, and F11 showed average *Q*_48_ values of 0.13 ± 0.04, 0.09 ± 0.01, and 0.06 ± 0.02 μg/cm^2^, respectively (Table 2). This corresponded to an average flux of 0.17 ± 0.05, 0.12 ± 0.02, and 0.09 ± 0.03 μg/cm^2^/h, respectively. Differences between *Q*_48_ and flux values obtained at 5% w/w (F9) and 20% w/w (F11) initial load were statistically significant (*P* = 0.026 and 0.041, respectively). Notably, increasing the initial FITC loading of NP stabilized with 1% w/v DMAB from 5% to 20% w/w was associated with an increase in particle size with a higher PDI for F11 and a decrease in zeta potential.

## 4 Discussion

The literature information provided proof of concept of enhanced transdermal delivery of drugs encapsulated in nanocarriers, particularly liposomes [9] and polymeric NPs [10] across MN-treated skin, promoting the transdermal delivery enhancing effect of either approach used separately. A better mechanistic insight is needed for optimization of this combined strategy for diverse drug delivery applications. At the outset, it could be postulated that the flux of a nanoencapsulated drug across MN-treated skin is a complex multifactorial process involving possible in-skin transport of the nanocarrier and the released drug through MN-created aqueous filled microchannels and deeper skin layers. For biodegradable polymeric NPs, assuming lack of transdermal delivery of the NPs across full thickness skin [10], [22], MN/nanoencapsulation-mediated skin permeation may be hypothesized to involve (a) translocation of drug-loaded NPs into MN-created channels, generating drug-rich reservoirs mainly in the epidermis and less densely in the dermis [22], a process influenced by MN array characteristics and application [10] in addition to interaction of NPs with the aqueous microchannel; (b) release of encapsulated drug from NPs, a process taking place mainly in microchannels [22] at a rate determined presumably by the physicochemical characteristics of NPs, the encapsulated drug and the physiological microenvironment; (c) diffusion of the released drug across hydrophilic deeper skin layers in series. The latter step is a concentration gradient-driven process, influenced by the drug molecular characteristics and impeded by diffusional resistances of the microchannels and the tissues beneath [20], [25].

In a recent study, we reported on the effect of MN array characteristics and application variables on the *in vitro* transdermal delivery of Rh B encapsulated in PLGA NPs across full thickness MN-treated porcine skin [10]. In the present work, we aimed at providing more knowledge on the contribution of characteristics of nanocarrier and encapsulated dye to MN-mediated transdermal delivery of nanoencapsulated dyes. The skin model used was full thickness porcine ear skin (approximately 1164 μm-thick), a well-established model representing full skin resistance and possessing characteristics similar to those of human skin [35].

### 4.1 Effect of NPs-related variables on skin permeation of the nanoencapsulated dye

Rh B or FITC-loaded NPs prepared at a relatively high emulsion homogenization speed (15,000 rpm) with 1% w/v DMAB were generally monodisperse with PDI < 0.2 and positively charged due to adsorption of the cationic surfactant. Zeta potential values exceeded 30 mV (36.1–67.6, Table 1), indicating physical stability [36]. This was obvious in TEM images of sample NPs (Fig. 3). FITC NPs prepared with PVA as emulsion stabilizer were negatively charged (−4.5 mV, Table 1).

#### 4.1.1 Effect of NPs size

Reduction in the particle size of 20% w/w Rh B-loaded PLGA 50:50 NPs (F1–F3) in the range 422.3–155.2 nm (Table 1) resulted in a significant increase in permeation of Rh B across MN-treated skin (Fig. 4). For instance, a 2.7-fold reduction in the mean diameter of F3 compared to F1 NPs led to a fivefold increase in *Q*_48_. It has been demonstrated that permeation characteristics of a NP through microchannels were significantly affected by NPs size relative to the pore size [37]. As the width of MN-created microchannels is usually in the micron range [23], that is, significantly larger than the size range of test NPs in the present study, and NPs size dependence of Rh B skin permeation can be explained by faster release of the encapsulated water soluble Rh B from smaller size NPs with larger surface to volume ratio. Particle size is a factor known to affect drug release from polymeric NPs [38]. Further, translocation of PLGA NPs across full thickness human abdominal skin was shown to be NPs size dependent, despite the larger microchannel size [22], [23]. Combined findings suggest deeper and more extensive influx of smaller NPs through MN-created channels leading to enhanced transdermal delivery of the water soluble dye released at the deeper NPs deposition sites. Consequently, factors related to particle size, such as wide particle size distribution and NPs aggregation potential, may affect not only NPs translocation across MN-treated skin but also transdermal delivery of nanoencapsulated hydrophilic permeants. This should be taken into consideration in the MN/nanoencapsulation modulation of skin permeation.

#### 4.1.2 Effect of NPs polymer composition and hydrophilicity

Increasing PLGA copolymer hydrophilicity by reducing the lactide to glycolide ratio (Table 1) significantly enhanced transdermal delivery of Rh B encapsulated in PLGA 50:50 NPs compared to PLGA 75:25 and 100:0 NPs of similar size, PDI, and zeta potential (Fig. 5 and Table 2). The results can be explained by greater compatibility of the more hydrophilic NPs with the aqueous milieu of microchannels, which reduces translocation resistance, enabling deeper penetration. The major diffusional resistance for a permeant traversing the skin through microchannels lies in the dermal layer [39]. Applying this principle to NPs means that reducing particle size and increasing hydrophilicity would enhance NPs movement through hydrophilic microchannels. Additionally, NPs with greater hydrophilicity will allow faster release of Rh B as a result of improved wettability of NPs and interstitial fluid penetration into the polymer matrix, a factor largely involved in drug release from polymeric-based delivery systems [40]. This was verified by the *in vitro* Rh B release data (Fig. 6). NPs with the three PLGA compositions (F4–F6) released Rh B at a hydrophilicity-dependent rate. Possible involvement of PLGA degradation in release enhancement is limited because of the relatively slow degradation rate of PLGA NPs [10].

#### 4.1.3 Effect on NPs surface charge

The effect of NPs charge type was investigated using 10% w/w loaded FITC NPs with positive and negative zeta potential (F10 and F12, respectively, Table 1). Despite the larger size, negatively charged NPs (F12, 367.0 nm, −4.5 mV) allowed significantly greater (*P* < 0.05) transdermal delivery of FITC compared to smaller NPs bearing a positive charge (F10, 122.0 nm, 57 mV) (Fig. 7). A 2.7-fold and 2.9-fold increases in *Q*_48_ and flux, respectively, could be observed (Table 2). A similar lag time suggested no change in the mechanism of drug transport. As porcine skin bears a net negative charge at physiological pH [41], repulsion of negatively charged NPs may reduce adsorption at its surface, driving NPs translocation deeper into the microchannels and enhancing flux of released FITC. These results are supported by the literature data [23] demonstrating faster diffusion of negatively charged fluorescent amine-modified polystyrene NPs (∼140 nm) through Isopore® membrane, a synthetic negatively charged membrane with cylindrical microchannels simulating microporated skin, compared to positively charged NPs. Results were explained by electrostatic repulsion between the negatively charged NPs and Isopore® membrane, preventing surface binding and accelerating the flow of NPs through aqueous channels.

Accordingly, surface charge type appears to have opposite effects on translocation of nanocarriers through microporated and intact skin, the latter being enhanced by a positive zeta potential that promotes electrostatic attraction with the negatively charged skin, enhancing penetration [42], [43], [44]. Further studies are needed to substantiate the NPs charge effects on permeation of nanoencapsulated molecules across deeper skin layers.

### 4.2 Effect of encapsulated dye-related variables on permeation

PLGA NPs with similar properties (50:50 PLGA composition, 57.0 mV zeta potential, 10% w/w dye loading) and close particle size (117.4 *versus* 122.0 nm for Rh B and FITC NPs, respectively, Table 1) were used as nanocarrier for Rh B and FITC to assess the contribution of encapsulated dye-related variables to skin permeation across MN-treated skin. The two dyes have different molecular characteristics in terms of chemical structure (a hydrophobic reactive S=C=N substituent in FITC structure, Fig. 1), MW (479.02 *versus* 389.38 Da for Rh B and FITC, respectively), and saturated solubility at physiological pH (0.99 *versus* 0.09 g/L for Rh B and FITC, respectively) [25]. Despite the similarity of the nanocarrier properties and a smaller MW (389.38 Da), significantly lower *Q*_48_ (97.0%) and flux (97.2%) values were obtained for FITC compared to the more soluble and larger MW Rh B (Fig. 8 and Table 2). This provided evidence for significant implication of the physicochemical properties of encapsulated molecules, particularly solubility, in the MN-mediated flux. Dye solubility would affect the release and molecular diffusion steps of the hypothesized mechanism. Higher solubility was reported to increase drug flux across MN-treated skin since the dermis does not represent a distinct barrier to hydrophilic drugs once the SC is bypassed [45]. For instance, Stahl et al. [46] demonstrated enhanced MN-driven permeation of the more hydrophilic permeants paracetamol and diclofenac compared to the lipophilic drugs ibuprofen and ketoprofen, irrespective of molecular weights. Further, enhanced transdermal flux was demonstrated for the water soluble hydrochloride form of naltrexone compared to the base [47] and the more soluble naltrexone glycolate compared to the hydrochloride salt [48]. The significantly lower flux of FITC can be ascribed to poor solubility due to the hydrophobic isothiocyanate substituent. This probably resulted in slower release from NPs and saturation of the microenvironment, resulting in reduced concentration gradient and molecular diffusion. In addition, the —N=C=S group was reported to enhance reactivity of FITC toward nucleophiles such as amine and sulfhydryl groups on proteins with the formation of covalent dye-protein conjugates *in vitro* [49] and interaction with biomacromolecules in the human skin [50].

Difference in skin permeation of Rh B and FITC was confirmed by confocal microscopic images obtained at 48 h post-skin treatment (Fig. 9a–d). These showed deposition of fluorescent Rh B and FITC NPs on the skin surface and probably superficial layers of *SC* in addition to infiltration of NPs inside MN-created channels (Fig. 9a and b, respectively), as reported previously [22]. Although both dyes in the free form can penetrate intact skin to a limited extent [19], [25], [51] and MN-treated skin to a larger extent [10], [25], contribution of the free form of the dyes to the observed fluorescence could be considered negligible in view of the limited dye release in the small volume of fluids at the skin surface. Noteworthy, FITC fluorescence was confined to microchannels (Fig. 9b), while diffuse Rh B fluorescence was clearly observed around the pores and more extensively in deeper skin layers (Fig. 9a). Depth penetration profiling demonstrated relatively deep Rh B permeation with detectable red fluorescence at 190 μm. On the other hand, the green FITC fluorescence was significantly reduced at a depth of 130 μm and almost disappeared at 150 μm (Fig. 9c and d, respectively).

Difference in permeation of Rh B and FITC was further substantiated by modulating the initial dye loading of NPs. While increasing Rh B loading (F6–F8, Table 1) generally resulted in a proportional significant increase in flux (Fig. 10), an increase in FITC loading (F9–F11) had an opposite effect (Fig. 10). Results verified the role of solubility as a primary determinant of the flux of small size permeants across hydrophilic deeper skin layers. Release of a larger amount of the water soluble Rh B dye around the NPs depot sites would build up a larger concentration gradient, the main driving force for transport of soluble permeants [20]. Increasing the concentration of hydrophilic permeants such as naltrexone salts resulted in increased MN-mediated transdermal flux [48]. Although data for more drugs are needed, drug loading of nanocarriers is a formulation factor that can be modulated to control permeation of nanoencapsulated drugs with different molecular characteristics through microporated skin for different skin delivery purposes.

Skin permeation data (Table 2) and CLSM imaging (Fig. 9) combined with absence of NPs in the receiver compartment during the study as confirmed by TEM provided sufficient evidence to suggest that only the free dye released from NPs permeated skin layers to the receiver compartment of the diffusion cell. It is worth mentioning that porcine skin barrier function proved to be maintained for 48 h using TEWL measurements [31] which was verified in this study by the absence of NPs in the receiver compartment after 48 h. Further, data indicated that post-infiltration of NPs in MN-created microchannels, a process affected largely by NPs characteristics, skin permeation rates of the released dyes were determined primarily by their molecular characteristics. The more hydrophilic Rh B dye permeated MN-treated skin at a significantly greater rate compared to the hydrophobic FITC dye of smaller MW, though both were encapsulated in PLGA NPs with similar properties. Findings tend to indicate that the MN/nanoencapsulation combined approach could be of benefit in enhancing transdermal delivery of hydrophilic drugs and controlling dermal localization of hydrophobic drugs.

## 5 Conclusions

Mechanistic insights into transdermal drug delivery using the dual microneedle (MN)/nanoencapsulation approach are provided based on a systematic study of the skin permeation of nanoencapsulated Rh B and FITC across MN-treated porcine skin. Intuitively, a mechanism hypothesized for this process should be based on integrated information regarding the translocation of polymer NPs as a charged colloidal system through micron-sized skin pathways and the molecular diffusion of the released dye in hydrophilic deeper skin tissues. Corroborated evidence obtained so far demonstrate the impact of NP characteristics such as size relative to microchannel dimensions, hydrophilicity, surface charge and potential NPs-skin interaction on both the skin translocation of NPs and the transdermal delivery of nanoencapsulated drug models. In addition to NPs composition and formulation attributes, molecular characteristics of the released molecule exert a significant impact on skin permeation. Poor solubility and potential interaction with skin constituents were shown to override molecular weight as impediments to transdermal delivery of the nanoencapsulated dye. Although further investigation with more drugs is needed to support findings of this study, it could be envisaged that synchronous optimization of the characteristics of MN array, nanocarrier and encapsulated agent would lead to improvement of the dual MN-nanoencapsulation strategy as an effective approach for transdermal and localized delivery of nanoencapsulated agents for diverse clinical applications such as enhanced vaccination and controlled steroid administration for eczema or psoriasis.
